# Regression of a Cardiac Calcified Amorphous Tumor Under Antiplatelet Therapy in a Patient With Ischemic Stroke: A Case Report and Literature Review

**DOI:** 10.7759/cureus.98077

**Published:** 2025-11-29

**Authors:** Soma Matsueda, Ryutaro Makino, Yushi Nagano, Nayuta Higa, Shunichi Tanaka, Hitoshi Yamahata, Ryosuke Hanaya

**Affiliations:** 1 Department of Neurosurgery, Graduate School of Medical and Dental Sciences, Kagoshima University, Kagoshima, JPN

**Keywords:** antiplatelet therapy, cardiac calcified amorphous tumor, central retinal artery occlusion, ischemic stroke, mitral annulus calcification

## Abstract

Cardiac calcified amorphous tumor (CAT) is a rare, non-neoplastic cardiac mass typically treated surgically. Spontaneous regression under antiplatelet therapy is uncommon. A 76-year-old woman presented with sudden visual loss in the left eye. Magnetic resonance (MR) imaging revealed small ischemic strokes in the left cerebellar hemisphere and right temporal lobe. Ophthalmological examination confirmed left central retinal artery occlusion. Transthoracic echocardiography (TTE) revealed mitral annulus calcification (MAC) and a mobile mass, raising suspicion for cerebral infarction due to cardiac CAT. Treatment with aspirin and heparin was initiated. On hospital day 10, brain MRI revealed a new asymptomatic infarction in the left frontal lobe, and surgical tumor resection was planned. The patient continued aspirin therapy while awaiting surgery. Follow-up TTE showed resolution of the CAT, and the patient was managed with aspirin alone. Because most CAT cases are treated surgically, reports of successful antithrombotic therapy are rare. CAT reduction or disappearance has been observed with oral aspirin, and its antiplatelet and anti-inflammatory effects may have contributed to tumor regression. Additionally, patients with MAC managed with anticoagulants are at elevated risk for calcification progression and recurrent stroke. In select patients with CAT and MAC, aspirin therapy may be considered with careful monitoring. Nonetheless, antithrombotic therapy has limitations and requires close monitoring, even after tumor regression.

## Introduction

Cardiac calcified amorphous tumor (CAT), first described by Reynolds et al. in 1997, is a non-neoplastic primary cardiac mass [1]. Histologically, CAT is characterized by nodular calcium deposits embedded in an eosinophilic, amorphous, fibrin-rich matrix, occasionally accompanied by chronic inflammation or surface fibrin [1-3]. Although histologically benign, CAT can appear intracavitary and pedunculated on imaging. This morphology may mimic primary cardiac neoplasms such as myxoma or fibroelastoma, which can result in misdiagnosis. Primary cardiac tumors are extremely rare, with a lifetime incidence of <0.02% [4]; CAT accounts for 2-3% of these cases [5]. CAT is typically detected on cardiac imaging, though it may first present clinically after an ischemic stroke. The conditions most frequently associated with CAT include mitral annulus calcification (MAC) (14%) and end-stage renal disease (21%) [2]. MAC is a chronic condition characterized by progressive calcification of the mitral valve annulus. It is more prevalent in the elderly, particularly in women. The presence of MAC has been associated with a two- to three-fold increased risk of stroke, possibly due to embolic events originating from the calcified annulus [6]. Herein, we report a rare case of acute ischemic stroke caused by CAT complicating MAC, in which the CAT spontaneously regressed under ongoing antiplatelet therapy, highlighting a potential non-surgical management approach.

## Case presentation

**Figure 1:**
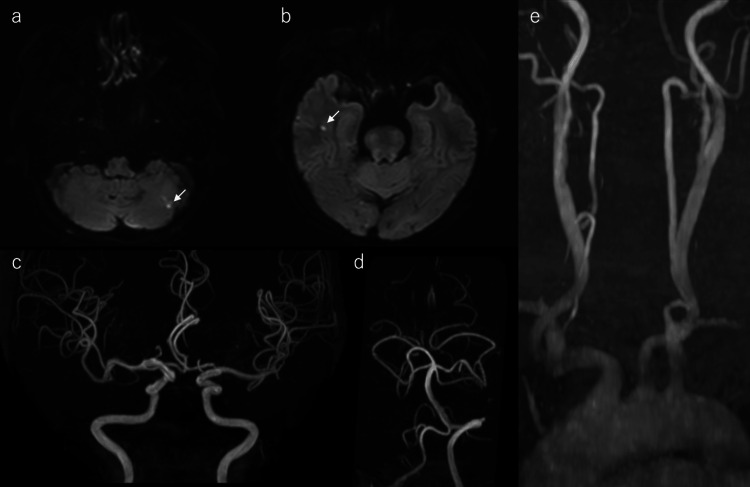
Magnetic resonance (MR) imaging Diffusion-weighted image demonstrated small ischemic infarcts in the left cerebellar hemisphere (white arrow) (a) and right temporal lobe (white arrow) (b). MR angiography revealed no significant stenosis or occlusion in the intracranial vessels (c, d) or carotid arteries (e).

A 76-year-old woman with a history of hypertension and hyperlipidemia presented with sudden visual impairment in the left eye and was transferred to our hospital. She was conscious and alert. The right pupil measured 3 mm, and the left pupil was dilated to 5 mm. The pupillary light reflex was present on the right but absent on the left. The heart rate was 80 bpm with a normal sinus rhythm. No neurological deficits were present other than visual impairment, and the National Institutes of Health Stroke Scale (NIHSS) score was 2 [7]. Laboratory tests showed a white blood cell count of 7.8×10^3^/µL, hemoglobin of 11.5 g/dL, and a platelet count of 255×10^3^/µL. Serum electrolytes and renal function were as follows: sodium 143 mEq/L, potassium 4.2 mEq/L, blood urea nitrogen 12.9 mg/dL, and creatinine 0.72 mg/dL. Liver function tests were within normal limits. Coagulation studies showed a prothrombin time-international normalized ratio (PT-INR) of 1.08, an activated partial thromboplastin time of 36.1 seconds, and a D-dimer level of 1.1 µg/mL. The lipid panel showed a total cholesterol level of 121 mg/dL, low-density lipoprotein (LDL) cholesterol 66 mg/dL, high-density lipoprotein (HDL) cholesterol 44 mg/dL, and triglycerides 81 mg/dL. The fasting glucose level was 114 mg/dL, the HbA1c was 6.1%, and the C-reactive protein level was 0.64 mg/L. Magnetic resonance (MR) imaging revealed small ischemic strokes in the left cerebellar hemisphere and right temporal lobe, respectively (Figures 1a, 1b). MR angiography showed no stenosis or occlusion of the intracranial or carotid arteries (Figures 1c-1e).

**Figure 2:**
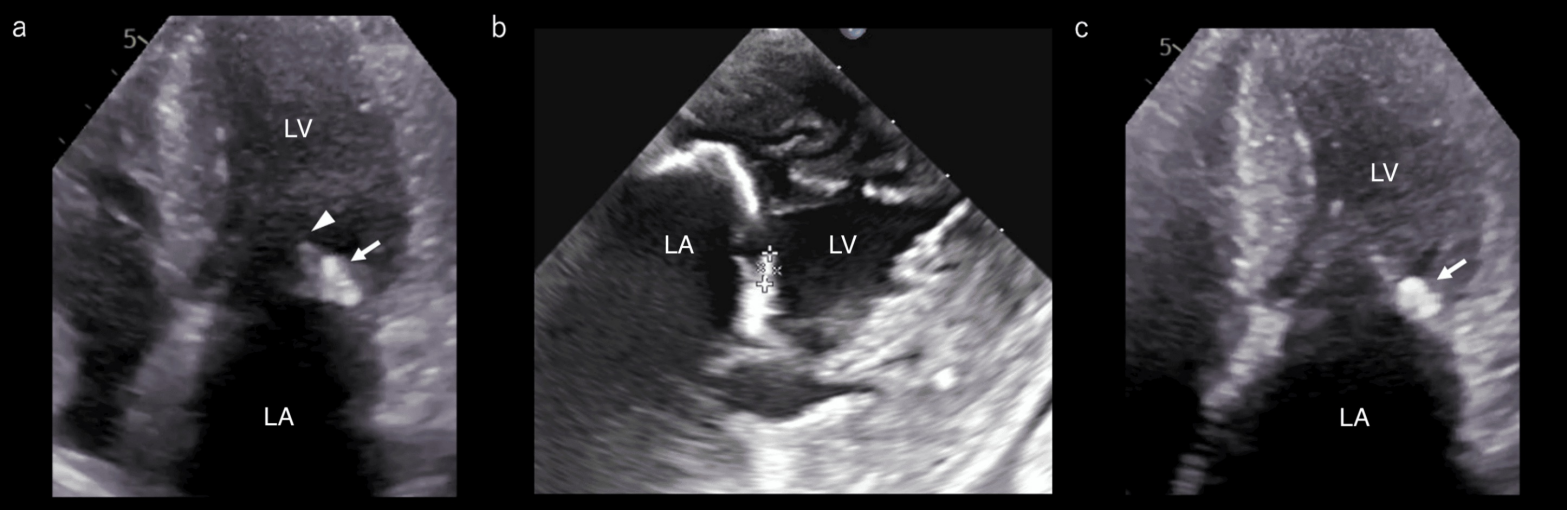
Transthoracic echocardiography (TTE) TTE on hospital day 2 shows mitral annulus calcification (MAC, white arrow), with a mobile mass at the same site (white arrowhead) (a). Transesophageal echocardiogram (TEE) on hospital day 4 confirmed the same findings, revealing a mobile mass measuring approximately 4 × 2 mm (b). Follow-up TTE on post-onset day 53 demonstrated resolution of the mobile mass previously adherent to the MAC (white arrow) (c). LA: left atrium; LV: left ventricle

The ophthalmologist diagnosed central retinal artery embolism based on the presence of inner retinal layer edema and a cherry-red spot. Treatment was initiated with 100 mg of aspirin and 10,000 units of heparin per day. There are no established guideline recommendations for antithrombotic therapy in cardiac CAT, and a workup for embolic sources was undertaken. On hospital day 2, transthoracic echocardiography (TTE) revealed calcification of the posterior mitral annulus, with a mobile mass at the same site (Figure 2a). Transesophageal echocardiography (TEE) on day 4 confirmed a mobile mass measuring approximately 4 × 2 mm (Figure 2b). The TEE showed no evidence of aortic thrombus. Continuous electrocardiographic monitoring during hospitalization and 24-hour Holter monitoring revealed no episodes of atrial fibrillation or other arrhythmias. Based on these findings, a CAT associated with MAC was suspected. No alternative embolic source was identified; thus, the cardiac lesion was considered the most likely embolic source. As there is no established evidence for antithrombotic therapy for CAT, heparin was discontinued on hospital day 9 in accordance with an Embolic Stroke of Undetermined Source (ESUS) based strategy, and aspirin monotherapy was continued. On hospital day 10, follow-up brain MRI revealed a new asymptomatic infarct in the left frontal lobe. Given the progression, a multidisciplinary discussion with cardiology and cardiovascular surgery concluded that surgical resection was warranted. As no neurological deterioration was observed, aspirin monotherapy at 100 mg daily was continued. Apart from persistent visual field impairment, the patient developed no new symptoms and was discharged on hospital day 27. Follow-up TTE on day 53 revealed resolution of the mass previously attached to the mitral annulus (Figure 2c).

After further multidisciplinary discussion, surgical resection was canceled in favor of continued observation. Although causality was uncertain, the potential therapeutic effect of aspirin was considered, and the medication was maintained. The patient was discharged home with an NIHSS score of 2 and a modified Rankin Scale (mRS) of 1 [7,8]. At follow-up, she remained functionally independent, with persistent left visual field impairment but no other neurological deficits. She continued aspirin therapy without interruption or complications. Follow-up TTE at six months confirmed sustained regression of the CAT, and MRI showed no new ischemic brain lesions.

## Discussion

Most previously reported cases of CAT-related cerebral infarction were managed surgically [9]. In total, antithrombotic therapy has been reported in eight CAT-associated stroke cases, including the present one (Table 1) [9-15].

**Table 1:** Summary of antithrombotic agents for ischemic stroke associated with cardiac calcified amorphous tumor (CAT) HT: hypertension; HL: hyperlipidemia; Af: atrial fibrillation; CHF: chronic heart failure; DM: diabetic mellitus; HD: hemodialysis; CKD: chronic kidney disease; TIA: transient ischemic attack; OP: operation

Post-onset antithrombotic agents	References	Age, sex	Comorbidity	Antithrombotic agents		Size of CAT		Surgery
				Pre-onset	Post-onset	First time	Follow-up	
Anticoagulants	Nishiguchi Y (2021) [9]	67, F	HT, HL	-	Anticoagulants (details unknown)	14×9.2 mm	-	Yes
	Singu T (2017) [15]	89, F	Af, HT, CHF, dementia	Warfarin	Warfarin	17.5x9.7 mm	Disappeared (11 days)	No
Antiplatelet therapy	de Hemptinne Q (2015) [10]	67, M	HT, HL	Aspirin clopidogrel	Aspirin clopidogrel	7x9 mm	-	Yes
	Morisaki A (2023) [11]	69, M	DM, pulmonary fibrosis	-	Antiplatelet therapy (details unknown)	22 mm	-	Yes
	Saito K (2016) [14]	70, M	HT, HD for CKD	-	Aspirin 200 mg	14×11 mm	10×9.8 mm (90 days)	No
	Present case	76, F	HT, HL	-	Heparin for 8 days, aspirin 100 mg	4×2 mm	Disappeared (53 days)	No
Both	Kimura M (2022) [13]	82, F	HT, TIA	-	Apixaban 5 mg, aspirin 100 mg	14.9×12.1 mm	13.8×9.7 mm (9 days)	Yes
	Kim H (2025) [12]	62, M	HT, DM, HL, HD for CKD	Clopidogrel	Heparin (pre-OP), warfarin, aspirin (post-OP)	25 mm	-	Yes

These comprised two cases treated with anticoagulants alone, four with antiplatelet therapy alone, and two with combination therapy. Given the limited evidence base, we reviewed both conservatively managed cases and those treated medically before surgery. In two cases, aspirin therapy was associated with CAT regression. One patient received preoperative apixaban 5 mg/day plus aspirin 100 mg/day, which resulted in lesion size reduction [13]. In another case, aspirin 200 mg/day led to tumor shrinkage and no stroke recurrence [14]. Several reports have also indicated that systemic infection may accelerate CAT progression [13,15].

Histopathological examination of CAT reveals amorphous fibrous tissue encasing multiple nodular calcifications, with lymphocytic infiltration and hemosiderin deposition within the fibrotic matrix, findings suggestive of an inflammatory component in CAT pathogenesis. In this case, as in prior reports, the antithrombotic and anti-inflammatory effects of aspirin may have contributed to tumor regression [14,16]. Previous studies have also reported that patients on warfarin exhibit higher incidences of mitral valve, aortic valve, and annular calcification compared to those not receiving warfarin [17,18]. Furthermore, MAC patients managed with anticoagulation demonstrate an elevated risk of recurrent stroke [6]. Considering the dual risks of valvular calcification and embolic recurrence in anticoagulated MAC patients, aspirin may be more suitable for secondary prevention in individuals with CAT coexisting with MAC. The literature review includes CAT cases in patients already on oral anticoagulants or antiplatelet agents. In one instance, a well-controlled patient on warfarin experienced CAT progression following an infection, with symptomatic improvement after antibiotic therapy [15]. Other cases involved patients receiving antithrombotic therapy as secondary stroke prevention [9,12]. Although most CATs are surgically excised, limited but emerging evidence supports the role of medical therapy in selected cases. Therefore, close clinical monitoring remains critical, even after imaging suggests tumor resolution. Surgical resection, while definitive, is invasive, and conservative treatment may be appropriate for asymptomatic or regressing lesions.

This study has some limitations. Histologic confirmation was not obtained, as the diagnosis was based solely on echocardiographic imaging. In a review of 41 histologically confirmed CAT cases, echocardiographic features were reported in 22; of these, 18 demonstrated high-intensity signals with partially hypointense or isointense areas, while four exhibited entirely high-intensity signals [15]. In the present case, the diagnosis was supported by high-intensity mobile echoes adherent to MAC on transthoracic and TEE. Further studies are needed to establish optimal treatment strategies, define indications for conservative management, and determine the appropriate duration of antiplatelet therapy in patients with CAT.

## Conclusions

We describe a MAC-associated CAT presenting with central retinal artery occlusion and multifocal ischemic stroke that resolved without surgery. Lesion regression coincided with antiplatelet therapy, and the antiplatelet and anti-inflammatory effects of aspirin may have contributed, though causality is uncertain. The diagnosis lacked histologic confirmation and relied on echocardiography. While surgery remains the standard of care, carefully selected patients with small, mobile lesions and no ongoing embolic events may be managed conservatively with close surveillance. Further studies are warranted.
